# Brown’s Valedictory Address

**Published:** 1856-06

**Authors:** 


### Dental Abstracts.

### BROWN'S VALEDICTORY ADDRESS.

The April number of the American Journal of Dental Science for 1856, contains among many other good articles the valedictory to the graduating class, by Solymon Brown, M. D., D. D. S., This is written in the Doctor's usual happy and humorous style, and contains much valuable instruction.

In relation to the advantages of a college course, the Dr. remarks:

The first American College of Dental Surgeons, which was also the first dental college of the world, at least during the present historic era, has, in like manner, been useful, honora ble and prolific. It has three living offspring, vigorous in health and honorable in renown; besides one that perished in its infancy as is sometimes the case with the *feeble* progeny of the human race.

As competition is said to be the life of trade, so mutual emulation and friendly rivalry, may, peradventure, contribute to the growth, the vigor and the permanency of all the dental colleges now in vogue. Some of them are situated very remotely from all others in the broad domain of our national confederacy, where states are like kingdoms in the old world; and consequently have ample scope, through diameters of hundreds of leagues, in a thickly populated country, for patronage and support.

The two well known facts that several dentists previously engaged in practice, have perfected their studies in these institutions, and returned to their business with redoubled popularity; and that all, or nearly all, the graduates of these colleges have been received with implicit confidence in the spheres of their practice, should induce many others of both these classes of individuals to follow their example. To a dental practitioner of either limited or unlimited preliminary advantages, and actuated by an honorable ambition, the outlay of time and money required for one or two annual courses in a dental college, would doubtless be twenty-fold repaid in future usefulness, reputation and emolument.

I am fully aware that the truth of these averments will be flatly denied by a large class of individuals who discover no personal advantage in either their acknowledgment or promulgation. But, let such persons be assured that, although they may never attain to the perspicacity of vision necessary to enable them to see, *in theory*, the truth of this doctrine, they will inevitably be implicated, *practically*, in all its issues, as surely as effects shall continue to follow causes.

If a hundred established practitioners of the younger class, and another hundred students who had never been in practice, were annually to throng the lectures and practical demonstrations of each of the dental colleges in our country, and there are none in any other land, this noble concourse would do no more than strict justice to itself, and to community, its patrons.

The practice of dentistry is destined to be elevated to higher usefulness than it has ever yet attained; and this can be effected only by the better education of its operators.

In relation to success the Dr. holds the following: We draw attention to the three last paragraphs, we have no doubt but many of the class felt and appreciated the force of the remarks.

We presume it came as near *poetry* as the Dr. was allowed to go.

But what is success? It is easy to define what it is not.

It is not necessarily nor generally, either worldly *wealth* or contemporary *fame*, which many seek so madly. If it were, the lives of many of the Cæsars and Napoleons of the world would not have been *failures*. For although the greatest of the Cæsars was compelled to borrow 40,000 sesterces, on his departure for the war in Spain, to place his private affairs in such a state, to use his own language, "as to make him worth just nothing at all;" that is, *pay his debts:* and although the first of the Napoleons, when departing for Italy, was unable to obtain credit of his *bottier* for a pair of military boots; yet both of these personages had unbounded contemporary fame, and controlled, at times, the riches of a continent.

All desirable *success* is the attainment of *useful ends* by *honest means*. And this most blessed boon, denied forever to self-aggrandizing and unscrupulous ambition, may be easily attained by every member of the class before me, with unerring certainty.

This desirable success *may* be indeed accompanied with both fame and fortune, as was the case with Washington; although the latter is rarely acquired by the mere labor of the hands, without adventurous speculations, either in "fancy stocks," or matrimonial alliances, both of which, as avenues merely to monetary riches will be avoided by the truly wise.

*Moderate gains*, attained by patient industry and prudent economy are all the worldly wealth of a pecuniary nature which dental practice promises to its most faithful devotees; and a settled conviction of this fact is indispensably necessary to their success. Nor is this to be regarded as matter of regret.

Enormous fortunes, like prodigious dinners, are generally burthens to their posessors. The *plethora* of either is not to be desired.

If four hundred dollars is considerably more than the portion of each individual in the common property of our country, supposing it apportioned alike to all, surely three or four thousand dollars per annum, is nothing less than *ample compensation* for the annual industry of any individual, inasmuch as this amount would absorb a portion of the general wealth equal to that of eight or ten of the non-producers, who are generally children, the aged and the infirm, the sick and the decrepid, to say nothing of the female half of the race, whose estimated earnings are classed in the columns of *duodecimals*.

Let moderation then, in all things, be your constant *safeguard* against the fatal reaction of blasted expectations. *Hope* being the last thing that dies in man, becomes his greatest oppressor when its cold remains lie lifeless on his heart. Hope, therefore, *sanely*, and be always contented with reasonable anticipations, and normal results.

But above all things else, learn, if not known already, that there are many things in life—even in mortal life—more valuable than fame or money, the attainment of which is man's *preeminent success*.

What will you say of a charming conjugal companion, the breath of whose love is sweeter than the gentle south wind whispering to a bed of violets?

What will you say of a group of laughing children, clambering upon your knees, hanging upon your lips, clustering about your neck, until you imagine the riches of the universe to be gathered at your fireside?

